# Function and Clinical Significance of Circular RNAs in Thyroid Cancer

**DOI:** 10.3389/fmolb.2022.925389

**Published:** 2022-07-22

**Authors:** Xuelin Yao, Qiu Zhang

**Affiliations:** Department of Endocrinology, First Affiliated Hospital of Anhui Medical University, Hefei, China

**Keywords:** thyroid cancer, circular RNAs, dysregulation, function, mechanism, perspective

## Abstract

Thyroid cancer (TC) is the leading cause and mortality of endocrine malignancies worldwide. Tumourigenesis involves multiple molecules including circular RNAs (circRNAs). circRNAs with covalently closed single-stranded structures have been identified as a type of regulatory RNA because of their high stability, abundance, and tissue/developmental stage-specific expression. Accumulating evidence has demonstrated that various circRNAs are aberrantly expressed in thyroid tissues, cells, exosomes, and body fluids in patients with TC. CircRNAs have been identified as either oncogenic or tumour suppressor roles in regulating tumourigenesis, tumour metabolism, metastasis, ferroptosis, and chemoradiation resistance in TC. Importantly, circRNAs exert pivotal effects on TC through various mechanisms, including acting as miRNA sponges or decoys, interacting with RNA-binding proteins, and translating functional peptides. Recent studies have suggested that many different circRNAs are associated with certain clinicopathological features, implying that the altered expression of circRNAs may be characteristic of TC. The purpose of this review is to provide an overview of recent advances on the dysregulation, functions, molecular mechanisms and potential clinical applications of circRNAs in TC. This review also aimes to improve our understanding of the functions of circRNAs in the initiation and progression of cancer, and to discuss the future perspectives on strategies targeting circRNAs in TC.

## Introduction

Thyroid cancer (TC) is the most common pervasive endocrine malignancy, especially in women (Kim et al., 2020). From 1990 to 2017, the incidence and mortality rates of TC has been increasing (Deng et al., 2020). In addition, the incidence and mortality rates of TC are still rapidly increasing, especially in many developed countries, with up to 586,202 newly diagnosed cases and 43,646 global deaths according to the estimates from Global Cancer Statistics in 2020 (Sung et al., 2021). By implementing early detection and optimal treatments, the survival rate of differentiated thyroid cancers (DTCs) has significantly improved. Patients diagnosed with early stage DTCs can achieve 5-years survival rates of approaching 98% and have a recurrence rate of less than 5–10% (Tuttle, 2018; Wang J. et al., 2020). However, the prognosis of patients with TC at an advanced-stage of the disease and multiple organ metastasis remains poor, with a 5-years survival rate of only 15.3% (Wang et al., 2014). Anaplastic TC (ATC), which accounts for 2% or fewer TC cases, is one of the most aggressive human malignancies and has a dismal prognosis with a median survival rate of less than 1 year (Keutgen et al., 2015; Yoo et al., 2019). To prolong the survival time and improve the quality of life of patients with TC, studies aiming to elucidate the tumourigenesis and molecular mechanisms of TC and to identify novel biomarkers and therapeutic targets for TC recurrence and metastasis are urgently needed.

Circular RNAs (circRNAs) are covalently closed single-stranded RNA molecules that have unique properties and powerful biological functions. In 1976, Sanger et al. first discovered single-stranded circRNA molecules in plant-based viruses (Sanger et al., 1976). Using electron microscopy, circRNAs have been identified in eukaryotes and humans as endogenous RNA (Hsu and Coca-Prados, 1979; Kos et al., 1986). However, circRNAs are mainly misinterpreted as non-functional products of pre-mRNA mis-splicing and only a few circRNAs (e.g. circSRY) are thought to have possible functions (Capel et al., 1993). In 2012, Salzman et al. found that circRNAs were the predominant transcript isoform in hundreds of human genes (Salzman et al., 2012). Subsequently, the identification and functional characteristics of ciRS-7 (also known as CDR1as), serving as the efficient miRNA sponges, formed a large class of post-transcriptional regulators (Hansen et al., 2013; Memczak et al., 2013). With the advancement of high-throughput RNA sequencing (RNA-seq) and bioinformatics algorithms, thousands of circRNAs have been identified to have tissue (Xia et al., 2017)/cell (Salzman et al., 2012)/development stage-specific (Chen B. J. et al., 2019) expression patterns in eukaryotes such as human, mice and zebrafish (Wesselhoeft et al., 2018). Several studies have been performed to explore the expression profiles of circRNAs in different cell types and diseases, and the outcomes have completely changed our view of circRNAs, which were originally thought to be junk by-products in the process of gene transcription (Goodall and Wickramasinghe, 2021). Numerous studies have focused on the potential role of circRNAs as promising disease biomarkers. Thousands of circRNAs have been identified as either oncogenes or tumour suppressors that mediate tumourigenesis, metastasis, and chemoradiation resistance in several cancers (e.g. TC, colorectal cancer, and renal cancer) (Hu Z. et al., 2020; Hanniford et al., 2020; Chen J. et al., 2021; Cen et al., 2021). In this review, we summarise the circRNAs involved in TC and their relevant clinical characteristics. A comprehensive understanding of circRNAs may provide valuable clues and useful information for future clinical applications of TC.

## Overviews of circRNAs

### CircRNA Biogenesis and Characteristics

**FIGURE 1:**
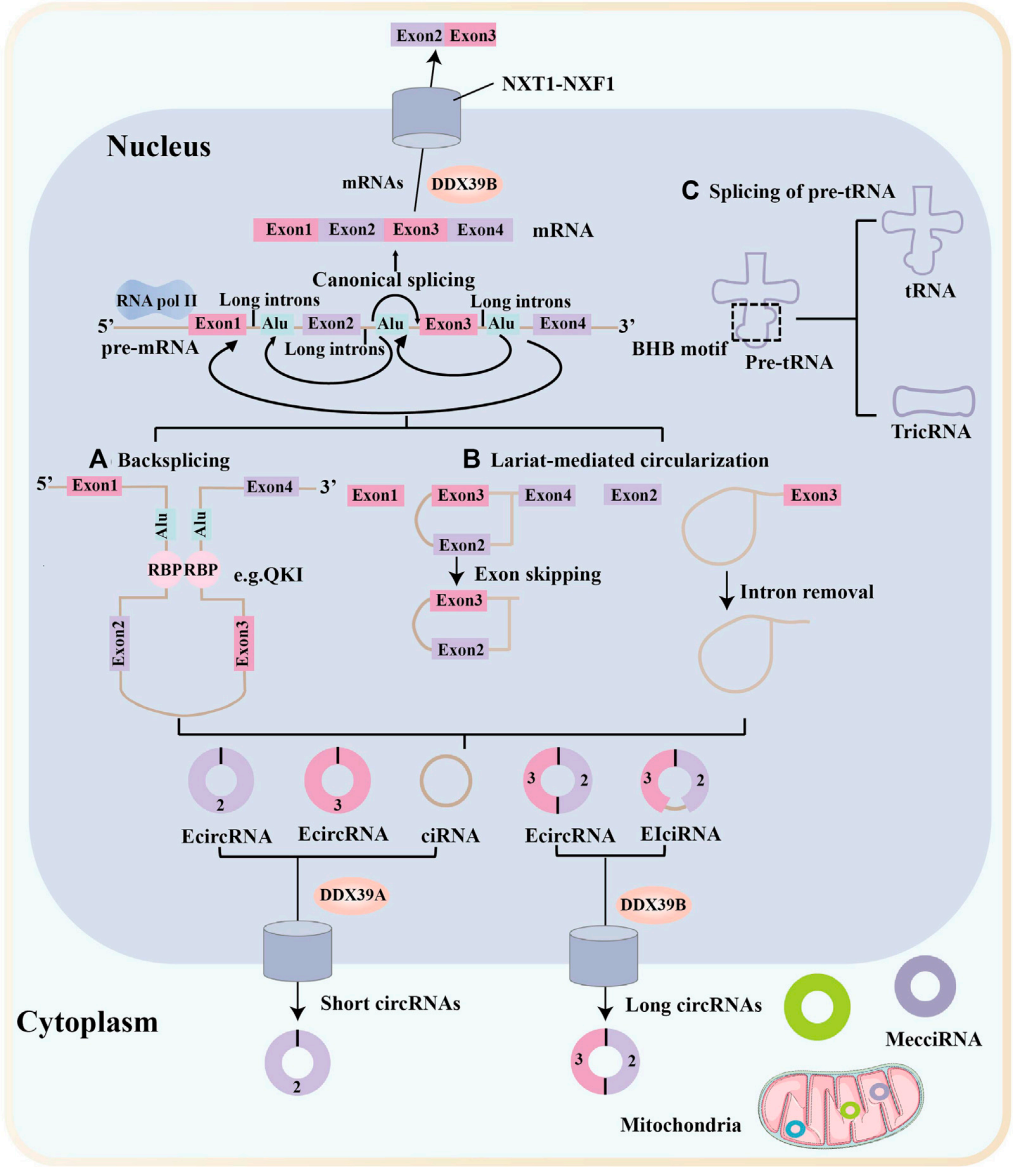
Biogenesis and nuclear export of circular RNAs. Messenger RNA (mRNA) synthesis occurs via canonical splicing, in which exons are aligned to generate the mRNA. Most circular RNAs (circRNAs) are transcribed by RNA polymerase II (Pol II) and formed by back-splicing of precursor mRNAs. Many circRNAs, especially those of low abundance, are formed as a result of base pairing between long flanking complementary introns containing inverted repeat elements, such as Alu repeats. **(A)** CircRNA biogenesis is fine-tuned by trans-acting RNA binding proteins (RBPs). **(B)** Another circRNA biogenesis model is the lariat-driven circularization, which occurs in exon-skipping events (left) or during intron removal from pre-mRNAs (right). **(C)** TricRNAs are another type of circRNA that are generated via the splicing of pre-tRNA. MecciRNAs are mitochondria-encoded circRNAs that are distributed in the mitochondria and the cytoplasm. Export of circRNAs from the nucleus require various proteins and occur in a length-dependent manner. DDX39B regulate the nuclear export of long circRNAs (>1,300 nucleotides), whereas DDX39A regulate the nuclear export of short circRNAs (< 400 nucleotides). NTF2-related export protein 1 (NXT1)-nuclear RNA export factor 1 (NXF1) heterodimeric export receptor recruit some complexes and release into the cytoplasm.

Most circRNAs are derived from known protein-coding genes with highly active promoters and consist of a single or multiple exons (Enuka et al., 2016). CircRNAs are primarily generated from primary transcripts through back-splicing (Figure 1A) and lariat-driven circularisation, which occurs in exon-skipping events (Figure 1B, left) (Barrett et al., 2015) or during intron removal from pre-mRNAs (Figure 1B, right) (Zhang et al., 2013). These models of circRNAs biogenesis are differ from the canonical linear splicing mechanism (Figure 1) (Li J. et al., 2020; Chen, 2020). Furthermore, circRNAs are resistant to degradation by exonucleases and are more stable than linear RNAs because of their covalently closed ring structures (Suzuki and Tsukahara, 2014). The most common circRNAs are exonic circRNAs (EcircRNAs), whereas the remaining circRNAs are intronic circRNAs (ciRNAs), exon-intron circRNAs (EIciRNAs), mitochondria-encoded circRNAs (MecciRNAs), and circRNAs of pre-tRNA splicing (TricRNAs) (Figure 1) (Chen, 2020).

Recent research into circRNA biogenesis has shown that back-splicing is catalysed by the canonical spliceosomal machinery and modulated by both intronic complementary sequences (ICSs) and RNA binding proteins (RBPs) (Li et al., 2018c). Pairing between ICSs on different introns is considered to bring the distal splicing sites closer, thereby enhancing back-splicing (Zhang et al., 2016). RBPs usually modulate back-splicing by directly connecting distal splice sites and binding to ICSs sites (Li et al., 2017; Okholm et al., 2020). For example, protein quaking (QKI) enhances circRNA formation by binding to its recognition motif in introns flanking circRNA-forming exons (Conn et al., 2015).

Similar to many linear mRNAs, circRNAs containing introns are frequently sequestered in the nucleus, but most circRNAs accumulate in the cytoplasm (Patop et al., 2019; Goodall and Wickramasinghe, 2021). A study by Huang et al. showed that a length-dependent evolutionarily conserved pathway mediated by DDX39B or DDX39A controls the nuclear export of circRNAs (Huang et al., 2018). Another study showed that the nuclear export of circNSUN2 was mediated by the m^6^A-binding protein YTHDC1, providing the first evidence that m^6^A controls circRNA translocation (Chen R.-X. et al., 2019).

### CircRNA Degradation and Exosomes Release

**FIGURE 2:**
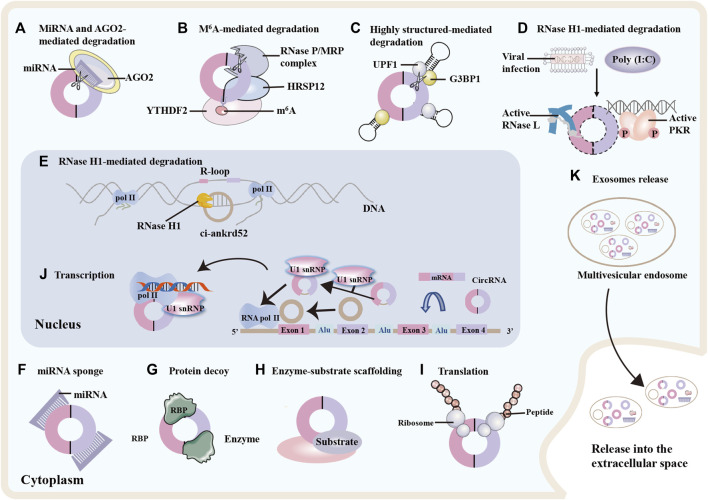
circRNAs degradation, functions and exosomes release. Degradation of circRNAs: **(A)** Specific microRNA (miRNA) direct binding to circular RNA (circRNA) facilitates cleavage of the circRNA by argonaute 2 (AGO2). **(B)** N^6^-methyladenosine (m^6^A)-modified circRNAs can be cleaved by the YTHDF2-HRSP12-RNase P/MRP complex. **(C)** The RNA-binding proteins UPF1 and its associated protein G3BP1 bind and regulate circRNA with highly-structured 3′UTRs, promoting their cleavage by the helicase activity of UPF1. **(D)** Upon viral infection, RNase L is activated and degrades the circRNAs, thereby releasing and activating protein kinase R (PKR) and decreasing global circRNA levels. **(E)** In the nucleus, RNase H1 degrades ciankrd52 dependent upon R-loop, thereby promoting RNA polymeraseII (Pol II) transcriptional elongation at ciRNA-producing loci. CircRNAs functions. **(F)** circRNAs can function as miRNA sponge by binding miRNA and inhibiting the expression of miRNA-mediated target messenger RNA (mRNA). **(G)** circRNAs can interact with RNA binding proteins (RBPs) and influence protein expression and function. **(H)** circRNAs can act as protein scaffolds for enzyme-substrate to modulate protein-protein interactions. **(I)** circRNAs with ribosome entry sites can be translated into proteins. **(J)** In the nucleus, circRNAs can interact with small nuclear ribonuleoprotein U1 (U1 snRNP) or promote Pol II elongation machinery to enhance RNA Pol II, then promoting gene expression. Exosomes release. **(K)** circRNAs can be enriched in exosomes and released into the extracellular environment upon exocytic fusion of multivesicular endosomes with the cell surface.

CircRNAs are resistant to degradation owing to their stable structure and the mechanisms underlying their degradation have only recently begun to be elucidated (Figure 2) (Li J. et al., 2020). Some circRNAs are degraded upon miRNA binding and argonaute-2 (AGO2) mediated cleavage (Figure 2A) (Hansen et al., 2011), whereas others are degraded by endoribonucleolytic cleavage by the endoribonuclease complex RNase P/MRP following modification with N^6^-methyladenosine (m^6^A) (Figure 2B). This degradation is mediated by the m^6^A reader protein YTHDF2 and adaptor protein HRSP12 in the cytoplasm (Park et al., 2019). Another decay mechanism involves ribonuclease L (RNase L) and double-stranded RNA-activated protein kinase R (PKR). Upon poly (I:C) stimulation or viral infection, RNase L is induced to degrade circRNAs, thereby releasing and activating PKR, which play a role in the early response of innate immunity (Figure 2D) (Liu C.-X. et al., 2019). Moreover, some RBPs are associated with the secondary structure of circRNAs. For example, upstream frameshift 1 (UPF1) and its associated endonuclease G3BP1 bind and unwind circRNAs, and the helicase activity of UPF1 leads to circRNA degradation (Figure 2C) (Fischer et al., 2020). Recently, it was reported that RNase H1 is responsible for nuclear circRNA degradation (Figure 2E) (Li et al., 2021c). This mechanism limits ciRNA accumulation by recruiting RNase H1 and resolves R-loops for transcriptional elongation at some GC-rich ciRNA-producing loci; one ciRNA, ciankrd52 with a locally open RNA structure, shows a stronger ability of R-loop formation and degradation by RNase H1 cleavage (Li et al., 2021c).

Some circRNAs are generally wrapped in multivesicular endosomes (40–160 nm) and secreted from various cells upon fusion of multivesicular bodies with the cell membrane (Figure 2K) (Kalluri and LeBleu, 2020; Seimiya et al., 2020). When using a stringent spliced reads per billion mapping cutoff, 1,428 of exosomal circRNAs and only 319 of cellular circRNAs were confirmed. This data suggest that the number of circRNAs in exosomes is on average higher than in the cancer cells from which they were released (Li Y. et al., 2015). Accumulating evidence indicates that exosomes play an important role in cancer progression, metastasis, and drug resistance (Xie et al., 2022; Yang et al., 2022). To date, exosomes have been detected in the plasma of patients with TC and carry biological effectors that contribute to the progression (Wu et al., 2019). Consequently, plasma exosomal circRNAs may be promising non-invasive biomarkers of TC.

### CircRNAs Functions

Based on the localisation of circRNAs, van Zonneveld et al. summarised and classified them into two categories: cytoplasmic circRNAs and nuclear-enriched circRNAs (van Zonneveld et al., 2021). The mechanisms of cytoplasmic circRNA function include (I) acting as miRNA and protein sponge (II) functioning as protein scaffolds, and (III) acting as a template for protein translation. Some circRNAs act as regulators of gene expression and have been identified in the nucleus (van Zonneveld et al., 2021).

Various cytoplasmic circRNAs have been reported to function as decoys for miRNAs and proteins, scaffolds for proteins, and templates for protein translation (Greene et al., 2017; Seimiya et al., 2020). To date, the most essential function of circRNAs is a miRNA sponge (Figure 2F). In the cytoplasm, some circRNAs serve as competing endogenous RNAs (ceRNAs), defined as miRNA sponges that block the regulation of miRNA on their target sites and affect gene expression and transcription regulation. For example, circLDLR behaves as a ceRNA sponge for miR-195-5p, resulting in a decreased miR-195-5p function and upregulated miR-195-5p target genes in papillary thyroid cancer (PTC) (Gui et al., 2020). Additionally, circRNAs often engage with numerous RBPs by acting as protein decoys (Figure 2G) and scaffolds (Figure 2H) to regulate protein functions and enhance the reaction kinetics of enzyme-substrate interactions (Du et al., 2016; Zang et al., 2020). For instance, circRNA_102,171 was found to promote the growth and invasion of PTC cells by binding to the β-catenin interacting protein 1 (CTNNBIP1) (Bi et al., 2018). In addition to protein sponging, circfoxo3 scaffolded p21 and cell cycle protein dependent kinase 2 to inhibit cell cycle progression (Du et al., 2016).

Recently, circRNAs (e.g., circZNF609) containing internal ribosome entry sites (IRESs) were found to be translated into proteins in eukaryotes (Chen et al., 2016; Legnini et al., 2017). In addition, some circRNAs serve as sources of pseudogene generation, modulating gene expression in the nucleus (Li Z. et al., 2015). For example, some nuclear EIciRNA (e.g., circEIF3J and circPAIP2) can enhance Pol II expression, thereby regulating gene expression at transcriptional and post-transcriptional levels (Figure 2J) (Li Z. et al., 2015).

To date, Numerous studies have shown the broad expression of endogenous circRNAs in all human tissues and circRNAs have been increasingly implicated in the regulation of cell proliferation, tumourigenesis, autophagy, neuronal functions and immune systems through various molecular mechanisms (Chen, 2020). However, biological functions have only been investigated for a minor fraction of the circRNAs identified to date, most of which still require further studies.

## CircRNAs Expression Profiles in TC

RNA-seq, circRNA-specific microarrays and bioinformatics analyses are the most commonly used methods for genome-wide profiling of circRNAs, and thousands of circRNAs have been identified in tissues, cells, exosomes, and blood of patients with TC (Table 1) (Peng et al., 2017; Hou et al., 2018; Lan et al., 2018b; Ren et al., 2018; Yang et al., 2019; Chu et al., 2020b; Guo et al., 2020a; Liu et al., 2020b; Liu Q et al., 2020; Long et al., 2020; Sun J. W. et al., 2020; Yang W. et al., 2020; Yang Y. et al., 2020; Yu et al., 2020; Chu et al., 2021; Guo et al., 2021; Li et al., 2021b; Lv et al., 2021; Qiu et al., 2021; Zhang et al., 2022). Hundreds of differentially expressed circRNAs (DECs) were identified between the tumour and non-tumour groups. For example, Peng et al. identified 453 circRNAs that were expressed in 6 matched PTC samples compared to control; 217 circRNAs were significantly upregulated, and 236 circRNAs were downregulated (Peng et al., 2017). Among four studies, the microarray dataset GSE93522 was the most commonly used database for secondary bioinformatic analyses intended to identify novel circRNAs for further research (Peng et al., 2017; Liu Q. et al., 2020; Li et al., 2021b; Qiu et al., 2021). Among these studies, circHACE1 was significantly downregulated (Li et al., 2021b) and hsa_circ_0004458 was upregulated in PTC tissues (Liu Q. et al., 2020). circ_0004053 and circ_0028198 was upregulated in PTC compared to that in normal samples (Qiu et al., 2021). Furthermore, researchers have focused on the differential expression and potential role of circRNAs in TC cell lines (Hou et al., 2018; Jiang et al., 2018; Long et al., 2020). Yu et al. detected 392 DECs between primary and lymph node metastasis (LNM) tumours, and of these DECs, circRNA-UMAD1 was selected as a sponge for miR-873 and was correlated with Gal3 levels in peripheral circulation (Yu et al., 2020). Exosomes have been reported to participate in intercellular communication by transmitting their cargo, including miRNAs, lncRNAs, proteins and even circRNAs to recipient cells, thereby regulating tumour progression (Zhou H. et al., 2021; Jafari et al., 2021). Yang et al. identified three differentially regulated circRNAs included hsa_circ_007,293, hsa_circ_031752, and hsa_circ_02013 in serum exosomes from patients with PTC compared with controls (Yang et al., 2019). These circRNAs (e.g., circFNDC3B) might be potential liquid biopsy indicators for the diagnosis of TC and may play regulatory roles in the progression of TC (Wu et al., 2020b).

**TABLE 1:** Expression profiling of circRNAs in thyroid cancer.

CircRNA (circBase ID or alternative titles based on the gene name or its position on a chromosome)	Samples		GEO database	Methods	Identi fied circR NAs	Differen tially express ed	Upregula ted circRNAs	Downreg ulated circRNAs	Ref.
CircRNAs that are upregulated (↑) in thyroid cancer samples compared to control									
1) hsa_circ_0061406 (circTIAM1)	Tissues		GSE168449	RNA-seq + qRT- PCR in 60 PTC and ANT	/	50	25	25	Zhang et al. (2022)
	PTC	ANT							
	3	3							
2) hsa_circ_0002360 (circRUNX1)	Tissues		/	RNA-seq + qRT- PCR in 52 PTC and ANT	/	100	100	/	Chu et al. (2021)
	PTC	ANT							
	3	3							
3) hsa_circ_0102272	Tissues		/	RNA-seq + qRT- PCR for 58 TC patients	/	54	35	19	Liu et al. (2020b)
	TC	ANT							
	5	5							
4) circRNA-UMAD1	Serum			RNA-seq	/	392	208	184	Yu et al. (2020)
	Invasive TC	TC							
	2	2							
5) hsa_circ_104566 (hsa_circ_0004458)	Tissues		GSE93522	Microarray + qRT-PCR for PTC and ANT samples	/	98	88	10	Peng et al. (2017)
6) hsa_circ_104565 (hsa_circ_0002111)	PTC	ANT							
7) hsa_circ_104595 (hsa_circ_0008016)	6	6							
8) hsa_circ_103110 (hsa_circ_0004771)									
9) hsa_circ_105038 (hsa_circ_0091894)									
10) hsa_circ_400064 (hsa_circ_0092315)									
11) hsa_circ_104268 (hsa_circ_0078738)									
12) hsa_circ_103307 (hsa_circ_0064557)	PTC	BTL							
13) hsa_circ_001379 (hsa_circ_0000516)	6	6							
14) hsa_circ_101356 (hsa_circ_0004846)									
15) hsa_circ_102002 (hsa_circ_0003505)									
16) hsa_circ_104433 (hsa_circ_0081342)									
17) hsa_circ_0000277	Tissues		GSE173299	Microarrays + qRT-PCR for 57 PTC and ANT patients	/	158	74	84	Guo et al. (2021)
18) hsa_circ_0074530	PTC	ANT							
19) hsa_circ_0057691	3	3							
20) hsa_circRNA000121	Tissues		/	Microarrays + qRT-PCR	/	690	400	290	Yang et al. (2020c)
21) hsa_circRNA051239	Invasive PTMC	PTMC							
22) hsa_circRNA001059	13	13							
23) hsa_circRNA102116									
24) hsa_circRNA000466									
25) circ_0004053	Tissues		GSE93522	Microarrays	/	137	115	22	Qiu et al. (2021)
26) circ_0028198	PTC	ANT							
	6	6							
27) hsa_circ_007293	Exosomes (serum)		/	Microarrays + qRT-PCR	/	22	3	19	Yang et al. (2019)
28) hsa_circ_031752	PTC	BTL							
	3	3							
29) chr20:20425608‑20472956‑	Tissues		/	RNA-seq + qRT- PCR in 45 PTC and ANT patients	/	53	45	8	Guo et al. (2020a)
30) chr5:161330882‑161336769‑	PTC	ANT							
31) chr7:22308338‑22318037	5	5							
32) hsa_circ_0082002									
33) hsa_circ_0002111									
34) hsa_circ_0008796									
35) chr7: 116695750–116700284+	Tissues		GSE171011	RNA-seq + qRT- PCR	16569	720	301	419	Lv et al. (2021)
36) chr7:116699071–116700284+	PTC	ANT							
37) chr5: 161330883–161336769−	4	4							
38) chr4: 25665378–25667298+									
39) chr1: 12578718–12579412−									
40) hsa_circ_0124055	Tissues		/	RNA-seq + qRT-PCR for 66 TC patients	/	231	133	98	Sun et al. (2020b)
41) hsa_circ_0101622	TC	ANT							
	5	5							
42) hsa_circ_0004458	Tissues		GSE93522	Microarrays and RNA-Seq	/	14	14	/	Liu et al. (2020d)
	PTC	ANT							
	6	6							
43) chr5:160757890-160763776−	Tissues		/	RNA-seq + qRT- PCR for 87 PTC patients	9103	87	41	46	Lan et al. (2018b)
44) chr12:40696591-40697936+	PTC	ANT							
45) chr7:22330794-22357656−	3	3							
46) chr21:16386665-16415895−									
47) hsa_circRNA_007148	Tissues		/	Microarrays + qRT-PCR for 40 PTC patients	/	383	206	177	Ren et al. (2018)
	PTC	ANT							
	3	3							
48) hsa_circ_406841	FRO cell line		/	Microarrays + qRT-PCR	/	50	25	25	Hou et al. (2018)
49) hsa_circ_00905	AGPS sh and KO groups compared with the control group								
50) hsa_circ_019252									
51) hsa_circ_089761									
52) hsa_circ_006050									
53) hsa_circ_074298									
54) hsa_circ_066556									
55) hsa_circ_101321									
56) hsa_circ_023016									
57) hsa_circ_019744									
CircRNAs that are downregulated (↓) in thyroid cancer samples compared to control									
1) hsa_circ_IPCEF1	Tissues		GSE173299	Microarrays + qRT-PCR for 57 PTC and ANT	/	158	74	84	Guo et al. (2021)
	PTC	ANT							
	3	3							
2) hsa_circ_0077514 (circHACE1)	Tissues		GSE93522	Microarrays + qRT-PCR	/	20	10	10	Li et al. (2021b)
	PTC	ANT							
	6	6							
3) hsa_circ_0007694	Tissues		/	qRT-PCR + RNA-seq in 3 PTC and ANT	/	129	87	42	Long et al. (2020)
	PTC	ANT							
	12	12							
4) hsa_circ_100777 (hsa_circ_0021553)	Tissues		GSE93522	Microarray + qRT-PCR for PTC and ANT samples	/				Peng et al. (2017)
5) hsa_circ_100395 (hsa_circ_0015278)	PTC	ANT				98	88	10	
6) hsa_circ_104348 (hsa_circ_0079891)	6	6							
7) hsa_circ_103454 (hsa_circ_0067103)	PTC	BTL				355	129	226	
	6	6							
8) hsa_circ_0020396	Tissues		GSE173299	Microarrays + qRT-PCR for 57 PTC and ANT patients	/	158	74	84	Guo et al. (2021)
9) hsa_circ_0095448	PTC	ANT							
10) hsa_circ_IPCEF1	3	3							
11) hsa_circ_0021549									
12) hsa_circRNA404686	Tissues		/	Microarrays + qRT-PCR	/	690	400	290	Yang et al. (2020d)
13) hsa_circRNA001729	Invasive PTMC	PTMC							
14) hsa_circRNA404686	13	13							
15) hsa_circRNA004183									
16) hsa_circRNA102051									
17) hsa_circRNA405571									
18) hsa_circ_020135	Exosomes (serum)		/	Microarrays + qRT- PCR	/	22	3	19	Yang et al. (2019)
	PTC	BTL							
	3	3							
19) hsa_circ_0072309	Tissues		/	RNA-seq + qRT- PCR in 45 PTC and ANT	/	53	45	8	Guo et al. (2020a)
	PTC	ANT							
	5	5							
20) chr5: 38481299–38530666	Tissues		GSE171011	RNA-seq + qRT- PCR	16569	720	301	419	Lv et al. (2021)
21) chr2: 159932176–159945082−	PTC	ANT							
22) chr10: 179994–249088+	4	4							
23) chr3: 121378716–121381532+									
24) chr1: 237423092–237445522+									
25) hsa_circ_0067934	Tissues		GSE93522	Microarrays and RNA-Seq	/	14	14	/	Liu et al. (2020d)
26) hsa_circ_0000673	PTC	ANT							
	6	6							
27) chr22:36006931-36007153−	Tissues		/	RNA-seq + qRT- PCR for 87 PTC patients	9103	87	41	46	Lan et al. (2018b)
28) chr7:91924203-91957214+	PTC	ANT							
29) chr2:179514891-179516047−	3	3							
30) chr9:16435553-16437522−	3	3							
31) hsa_circRNA_047771	Tissues		/	Microarrays + qRT-PCR for 40 PTC patients	/	383	206	177	Ren et al. (2018)
	PTC	ANT							
	3	3							
32) hsa_circ_404686	FRO cell line		/	Microarrays + qRT-PCR	/	50	25	25	Hou et al. (2018)
33) hsa_circ_00367	AGPS sh and KO groups compared with the control group								
34) hsa_circ_001729									
35) hsa_circ_004183									
36) hsa_circ_100790									
37) hsa_circ_104270									
38) hsa_circ_102049									
39) hsa_circ_406494									
40) hsa_circ_100787									
41) hsa_circ_082319									

Abbreviations: PTC:papillary thyroid cancer, ANT: adjacent non-tumor tissue, BTL:benign thyroid lesion, PTMC: papillary thyroid microcarcinoma, qRT-PCR: quantitative reverse transcriptase-polymerase chain reaction, RNA-Seq: RNA sequencing. AGPS: alkylglycerone phosphate synthase, sh: short hairpin, KO:knockout.

## Biological Functions of circRNAs in TC

### Oncogenic Activity of circRNAs

**FIGURE 3:**
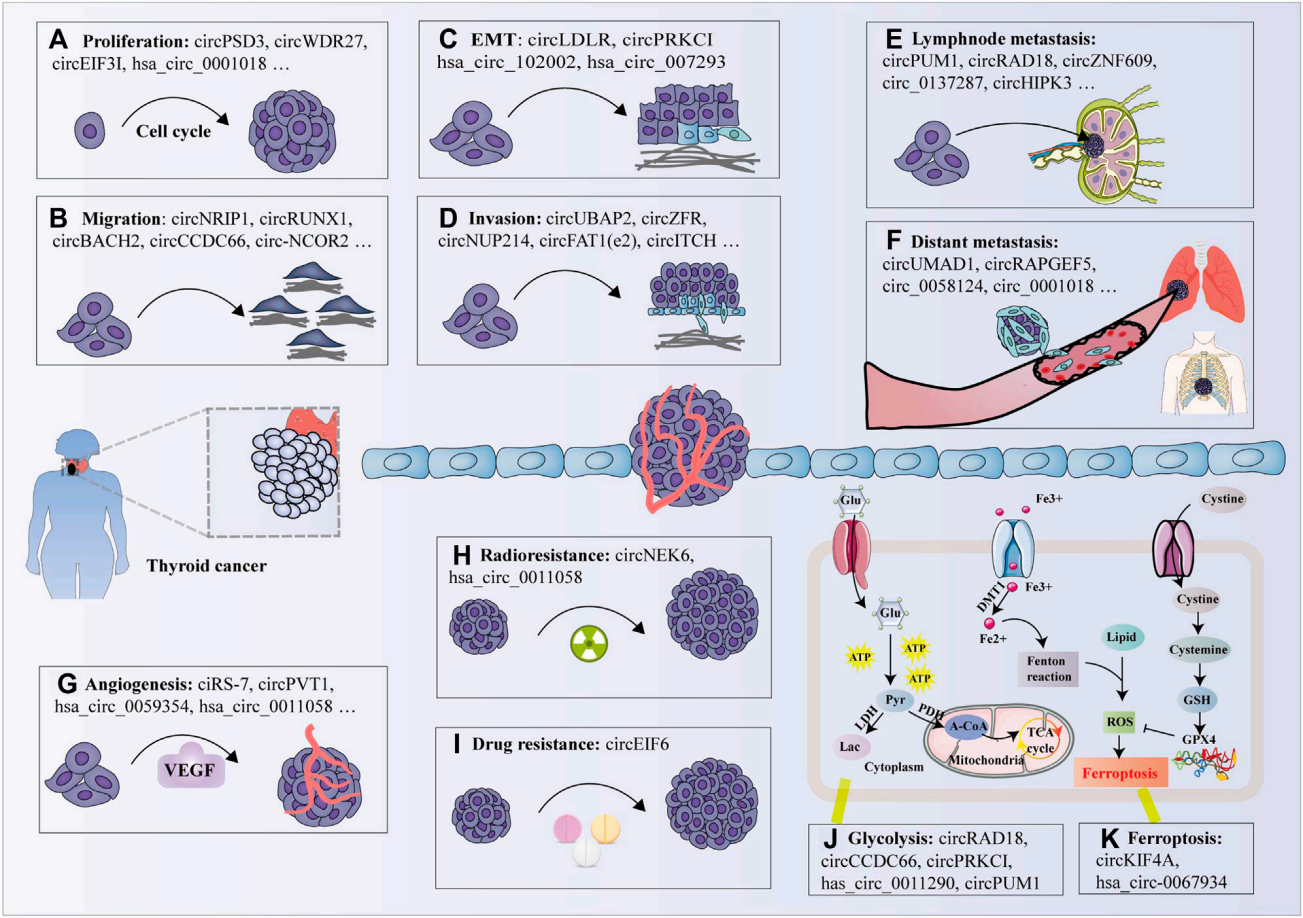
Biological functions of circRNAs in thyroid cancer. **(A)** circRNAs promote cell proliferation by promoting (e.g., circPSD3) or inhibiting (e.g., circITCH); **(B)** circRNAs promote cell migration by facilitating (e.g., circNRIP1) or inhibiting (e.g., hsa_circ_0007694); **(C)** circRNAs modulate the epithelial-mesenchymal transition (EMT) process by promoting (e.g., circLDLR); **(D)** Some circRNAs promote cell invasion (e.g., circZFR), while other circRNAs inhibit cell invasion (e.g., circNEURL4); **(E)** Several circRNAs are correlated with lymphnode metastasis (e.g., circPUM1); **(F)** A few circRNAs are associated with distant metastasis (e.g., circUMAD1); **(G)** Some circRNAs have been shown to promote tumor angiogenesis (e.g., ciRS-7) by modulating vascular endothelial growth factor A (VEGFA) expression; **(H)** Several circRNAs facilitate radioresistance in TC cells (e.g., circNEK6); **(I)** Individual circRNA promotes the drug-resistance of TC cells (e.g., circEIF6); **(J)** Certain circRNAs modulate glycolysis (e.g., circRAD18); and **(K)** ferroptosis (e.g., circKIF4A) in thyroid cancer cells. Glu: glucose, ATP: adenosine triphosphate, Pyr: pyruvate, Lac: lactate, LDH: lactate dehydrogenase, PDH: pyruvate dehydrogenase, A-CoA: acetyl-CoA, TCA cycle: tricarboxylic acid cycle, GSH: glutathione, ROS: reactive oxygen species, GPX4: glutathione peroxidase 4.

A growing body of evidence has confirmed that upregulated circRNAs function as oncogenes involved in the occurrence and progression TC by regulating malignant cell phenotypes, including cell colony formation, proliferation, migration, invasion and epithelial-mesenchymal transition (EMT) (Supplementary Material S1, Figure 3).

To date, numerous circRNAs have been proposed to bind to various miRNAs and inhibit their mRNA activity via a function known as miRNA spongs or decoys (Supplementary Materials S1,2). Studies suggested that identical circRNAs contain multiple miRNA-binding sites that can perform various functions by sponging different miRNAs and inhibiting their mRNA activity (Qi et al., 2021b), as exemplified by circPSD3, which contains target sites for miR-7-5p, miR-885-5p and miR-637 (Jin et al., 2018; Li et al., 2021e; Zhu et al., 2021). Studies have also revealed that different circRNAs contain the same type and miRNA binding sites that can specifically bind to miRNAs, thereby reducing miRNA activity and upregulating the expression of miRNA-related target genes (Shu et al., 2020; Luo et al., 2021), such as hsa_circ_0058124 and circUBAP2, which are revealed as miR-370-3p sponges and promote proliferation and invasion of TC cells (Liu L. et al., 2020; Xiong et al., 2021b). In addition, specific circRNAs protect homologous mRNAs from miRNA-mediated degradation by inhibiting miRNA activity (Zeng et al., 2021). For example, Zeng et al. revealed that circPVT1 serves as a ceRNA to sequester miR-195 and promote the PVT1-mediated malignant progression in PTC (Zeng et al., 2021). Accumulating evidence has identified individual circRNAs containing multiple RBP motifs, suggesting circRNAs may sponging protein and modulate RBP-dependent functions (Bronisz et al., 2020; Tsitsipatis et al., 2021). For instance, circ_102171 has been shown to accelerate the malignant behaviour of PTC cells by interacting with CTNNBIP1 and regulating the Wnt/β-catenin signalling way. Silencing of circ_102171 suppressed PTC cell proliferation, migration and invasion, while promoting apoptosis *in vitro* and inhibiting PTC growth *in vivo* (Bi et al., 2018). In addition, certain circRNAs promote angiogenesis in TC (Li S. et al., 2020; Zeng et al., 2021). A tube formation assay showed that circ_0011058 knockdown notably decreased fibroblast growth factor 2 and vascular endothelial growth factor A, which are important activators of angiogenesis, thereby impeding the proliferation and angiogenesis of PTC cells (Zhang Z. et al., 2021).

### Anti-Tumour Activity of circRNAs

Generally, six downregulated circRNAs (e.g., circHACE1, circITCH, circNEURL4, hsa_circ_100395, hsa_circ_0007694, circSH2B3) function as tumour suppressors in TC, inducing cell cycle arrest and apoptosis while hampering cell proliferation, migration, and invasion (Peng et al., 2017; Wang M. et al., 2018; Long et al., 2020; Ding W. et al., 2021; Li et al., 2021b; Sa et al., 2021) (Supplementary Materials S1,S2, Figure 3). In addition, recent studies have identified that several upregulated circRNAs are involved in cell signal transduction, which is a process of transferring molecular signals from the extracellular space into the cell through the cell membrane, thereby inducing the tumourigenesis of PTC (Zhou et al., 2018; Yao et al., 2019). For example, Wang et al. observed that circ-ITCH overexpression significantly inhibited the proliferation and invasion of PTC cells by upregulating the expression of CBL and promoting apoptosis *in vitro*, which led to suppression of the Wnt/β-catenin pathway and the tumour-suppressive role of circ-ITCH (Wang M. et al., 2018).

### Modulating Radioresistance

Radioactive iodine (RAI) is used after thyroidectomy to ablate the residual normal thyroid remnant, as adjuvant therapy, and to treat TC (Luster et al., 2014). Problematically, it has been reported that approximately 30% of advanced DTC will eventually lose the ability to concentrate radioiodine and dedifferentiate due to decreased expression of Na/I symporter (NIS) (Trouttet-Masson et al., 2004; Woodrum and Gauger, 2005). Reestablishing the ability to concentrate iodine and redifferentiation becomes the principal problem faced by the radioactive iodine therapy for poorly DTC. Accumulating evidence suggests that some circRNAs play increasingly important roles in the regulation of radioaction responses (Supplementary Material S1, Figure 3H) (Gu et al., 2021; Wu et al., 2021). For example, Chen et al. observed that circ_NEK6 expression was elevated in ^131^I-resistant DTC tissues and cell lines, and knockdown of circ_NEK6 repressed ^131^I resistance in DTC and suppressed cell proliferation, migration, and invasion abilities while inducing cell apoptosis and DNA damage (Chen F. et al., 2021). Furthermore, Sa et al. first identified that downregulation of circSH2B3 increased ^125^I uptake and NIS expression levels in PTC cells treated with aryl hydrocarbon receptor (AhR) antagonists, which has been implicated in the dedifferentiation of radioiodine-refractory PTC (Sa et al., 2021). These results support the evidence that circRNAs directly or indirectly mediate radioresistance by forming a ceRNA network and may function as therapeutic targets to improve the efficacy of refractory/relapsed patients.

### Regulating Chemoresistance

Drug treatment together with surgical operation, radiotherapy and biotherapy constitute the main approaches to cancer treatment (Jaaks et al., 2022). With the clinical application of anti-tumour molecular targeting drugs, the survival rate of patients with tumours have been significantly extended (Stone et al., 2017). However, chemoresistance remains an intractable problem that hinders better patient prognosis (Herzog et al., 2021; Lampropoulou et al., 2022). Accumulating evidence suggests that ncRNAs, including miRNAs, lncRNAs, and circRNAs, may drive drug resistance in various cancers, including TC (Supplementary Material S1, Figure 3I) (Liu F. et al., 2018; Gao et al., 2020; Zhang H. et al., 2021; Lampropoulou et al., 2022). Liu et al. showed that hsa_circ_0060060 (circEIF6) overexpression was negatively correlated with miR-144-3p and enhanced cisplatin resistance by autophagy activation in TPC1 and BHT101 cells, suggesting that circEIF6 plays a crucial role in cisplatin resistance (Liu F. et al., 2018). Currently, studies of circRNAs in chemoresistance are rare, and further investigations are needed to explore the detailed mechanisms and potential clinical applications.

### Regulation of TC Metabolism

Deregulated metabolism, which is widespread in tumor progression, provides an essential source for proliferation and growth of cancer cells. Glycolysis, fatty acid oxidation, and amino acid metabolism are responsible for metabolic reprogramming of cancer cells (Stine et al., 2022). Under adequate oxygen condition, cancer cells increase glucose uptake and ATP and lactic acid accumulation through glycolysis. This phenomenon is termed as aerobic glycolysis or the Warburg effect (Warburg et al., 1927). Some circRNAs promote the Warburg effect and regulate the malignant behaviour of many tumours by sponging miRNAs (Chen X. et al., 2019; Cao et al., 2020). Targeting the intrinsic metabolism of cancer cells has proven to be a promising therapeutic strategy for TC (Supplementary Material S1, Figure 3J) (Liu Y. et al., 2021; Zhang Q. et al., 2021). Pyruvate dehydrogenase kinase (PDK) is a critical modulator of key glycolysis enzymes and is associated with EMT, poor prognosis and therapy resistance (Atas et al., 2020). A recent study confirmed that silencing circRAD18 remarkably inhibited cell glucose uptake, lactate production and the expression level of PDK1 protein in PTC cells, indicating the regulatory effect of circRAD18 on glucose metabolism reprogramming in PTC (Chen et al., 2021e). Consistent with these findings, knockdown of circCCDC66 suppressed the glycolytic metabolism of TC by targeting the miR-211-5p/PDK4 axis (Ren et al., 2021). Moreover, alterations in fatty acid metabolism can influence energy storage, affect drug resistance, modulate cell proliferation and survival, and stimulate the extracellular environment (Röhrig and Schulze, 2016). Wen et al. identified four recurrence-related genes (*PDZK1IP1*, *TMC3*, *LRP2* and *KCNJ13*) and established a four-gene signature recurrence risk model, indicating that lipid metabolism-related gene profiling represents a potential marker for prognosis and treatment decisions for PTC patients (Wen S. et al., 2021). Nevertheless, the mechanism of circRNAs in lipid metabolism of TC remains largely unknown and is expected to become a novel field in the study of circRNAs in TC.

### Function of circRNAs in Ferroptosis and Other Mechanisms

Ferroptosis is an iron- and reactive oxygen species (ROS)-dependent form of cell death, characterised mainly by cytological changes (Huang et al., 2021). Accumulating evidence suggests that circRNAs may function as essential regulators of ferroptosis in cancers, including TC (Supplementary Material S1, Figure 3K) (Wang H.-H. et al., 2021; Chen et al., 2021d; Yang et al., 2021). For example, Wang et al. observed that silencing circ_0067934 increased the levels of ferroptosis-related markers, including Fe^2+^, iron, and ROS, in TC cells, suggesting that circ_0067934 may serve as a potential therapeutic target by regulating ferroptosis for the treatment of TC (Supplementary Material S1, Figure 3K) (Wang H.-H. et al., 2021). In addition, individual circRNAs may modulate the expression of apoptosis-related proteins (e.g., Bax and caspase-3) (Xia et al., 2020), metastasis-associated protein (MTA2, MTA) (Yang Y. et al., 2020; Luan et al., 2020), and epithelial mesenchymal phenotype biomarkers (MMP2, MMP9, Twist1, E-cadherin, N-cadherin, vimentin, and Slug) (Han J.-y. et al., 2020; Gui et al., 2020; Xia et al., 2020; Zhang W. et al., 2021; Wang W. et al., 2021) to mediate cell apoptosis, metastasis, and EMT. In addition, a few circRNAs may indirectly activate or inactivate several vital signaling pathways by suppressing miRNAs, such as the NOTCH3/GATAD2A (Yao et al., 2019), JAK/STAT/AMPK (Cui and Xue, 2020), PI3K/AKT/mTOR (Long et al., 2020), and Wnt/β-catenin signalling pathways (Bi et al., 2018; Chen et al., 2018; Long et al., 2020; Zeng et al., 2021) For instance, Cui et al. observed that hsa_circ_100,721 (circDOCK1) serves as a ceRNA for miR-124, leading to dampening signal transduction of the JAK/STAT/AMPK pathway (Cui and Xue, 2020). Dong et al. revealed that circ_0067934 acts as a molecular sponge for miR-1301-3p to induce malignant effects in PTC cells, resulting in the activation of PI3K/Akt and MAPK pathways (Dong et al., 2022). However, the specific mechanisms underlying these circRNA functions remain unknown and require further study.

## Potential Application of circRNAs in TC

At around the time when circRNAs were first discovered, Sanger et al. described circRNAs as viroids with pathogenic activity towards certain higher plants (Sanger et al., 1976). However, with in-depth studies of circRNAs, increasing evidence has emphasised that circRNAs are essential for gene expression. CircRNAs are highly abundant and widely distributed in nearly all types of human tissues, cells, and bodily fluids, such as blood (Chen C. et al., 2022), bile (Xu et al., 2021), saliva (Jafari Ghods, 2018), breast milk (Zhou Y. et al., 2021), urine (He et al., 2021), ascites (Du et al., 2022), pleural effusion (Wen et al., 2018), synovial fluid (Wu et al., 2022), cerebrospinal fluid (Wang Z. et al., 2022), and bronchoalveolar lavage fluid (Liu Q.-P. et al., 2021), and are even enriched in exosomes (Fan et al., 2022).

CircRNAs account for approximately 1% of poly(A) RNA in human cells (Jeck and Sharpless, 2014), and over 25,000 distinct circRNAs have been identified in human fibroblasts (Jeck et al., 2013). CircRNAs are prone to detection because of their higher expression in peripheral whole blood compared to linear ncRNAs (Memczak et al., 2015). In addition, circRNAs are resistant to RNase R digestion and can be easily detected using quantitative reverse transcription-polymerase chain reaction (qRT-PCR) assays (Chen L. et al., 2022). Finally, their expression levels are extremely diverse and variable based on the cell type and development stage of the tissues (Chen, 2020). With the advantages detailed above, numerous circRNAs can be characterised as non-invasive and repeatable biomarkers. Here, we used a few typical examples to discuss the clinical implications of specific circRNAs in TC.

### CircRNAs as Promising Diagnostic and Prognostic Biomarkers for TC

**FIGURE 4:**
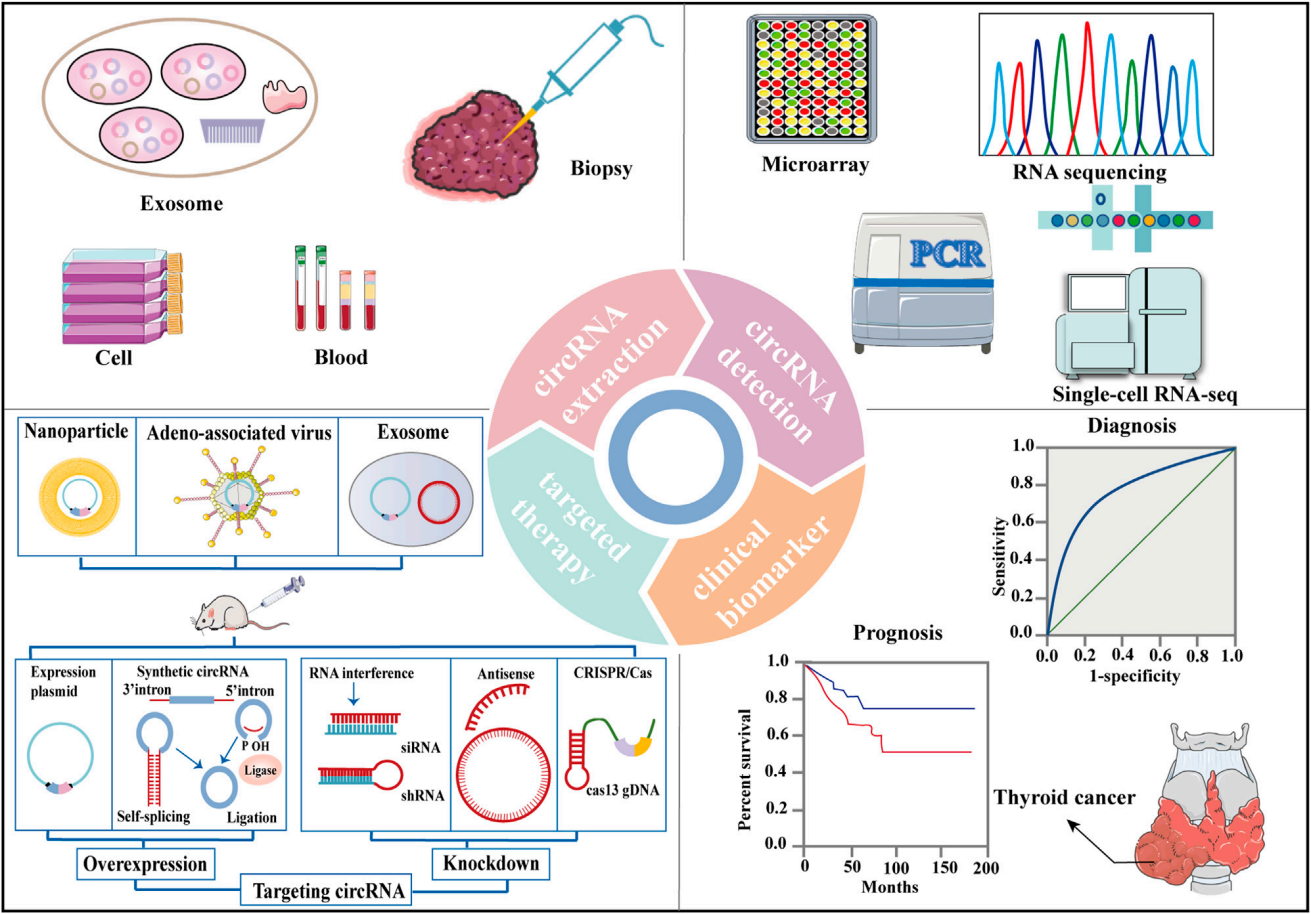
Clinical applications of circRNAs in thyroid cancer. CircRNAs are extracted from cell, blood, biopsy and exosome from TC patients. RNA sequencing, microarray, single-cell RNA-seq, quantitative reverse transcriptase-polymerase chain reaction (qRT-PCR) assays are methods that can be used to detect circRNA levels. CircRNAs are significantly associated with many clinicopathologic characteristics of TC and with TC patient survival parameters, rendering them potential diagnostic and prognostic biomarkers for TC. Overexpression or knockdown of target circRNAs resulting from delivery may serve as potential therapeutic approaches for TC.

Compared with normal controls, circRNAs present significantly differential expression profiles in TC tissues and blood from patients with TC; thus, they are regarded as promising and ideal candidates for the diagnosis of TC owing to their abnormal expression and high specificity (Table 2; Figure 4) (Jin et al., 2018; Lan et al. 2018a; Ren et al. 2018; Wang et al. 2018b; Wei et al. 2018; Cai et al. 2019; Yao et al. 2019; Wang et al. 2019a; Fan et al. 2020; Han et al. 2020a; Hu et al. 2020b; Liu et al. 2020b; Shi et al. 2020; Sun et al. 2020b; Wang et al. 2020b; Wu et al. 2020a; Xue et al. 2020; Ye et al., 2020; Yu et al., 2020; Zhang et al. 2020b; Chu et al., 2021; Ding et al. 2021a; Ding et al. 2021b; Du et al. 2021; Guo et al. 2021; Li et al. 2021a; Li et al. 2021b; Li et al. 2021d; Lin et al. 2021; Liu et al. 2021b; Luo et al., 2021; Ma and Kan, 2021;Qi et al., 2021b; Xiong et al., 2021b; Zeng et al., 2021; Zhang et al. 2021c; Zhang et al. 2021d; Zheng et al. 2021a; Zhu et al., 2021; Dong et al., 2022; Li et al. 2022; Nie et al. 2022). For example, Zhang et al. documented significant upregulation of circRNA_103,598 expression in PTC tissues and cell lines, with an area under the receiver operating characteristic (ROC) curve (AUC) as high as 0.9456 (Zhang S. et al., 2020). Sun et al. demonstrated that two circRNAs (hsa_circ_0124055 combined with hsa_circ_0101622) provided a more powerful diagnostic value (AUC = 0.911, 95% CI: 0.859–0.962, *p* < 0.001) than the use of hsa_circ_0124055 (AUC = 0.836) or hsa_circ_0101622 (AUC = 0.805) alone (Sun JW. et al., 2020).

**TABLE 2:** Clinical significance of dysregulated circRNAs in thyroid cancer.

Circular RNAs	Associated clinicopathological characteristics	Diagnostic value	Prognostic value	Ref.
CircRNAs that are upregulated (↑) in thyroid cancer samples compared to control				
1) circRUX1	tumour size, lymphnode metastasis, TNM stage, extrathyroidal extension	/	/	Chu et al. (2021)
2) hsa_circ_0004458	tumour size, lymphnode metastasis, TNM stage, distant metastasis	/	/	Jin et al. (2018)
3) ciRS-7	tumour size, lymphnode metastasis	/	/	Han et al. (2020a)
4) circEIF3I	tumour size, lymphnode metastasis, TNM stage	/	/	Wang et al. (2020b)
5) circPSD3	tumour size, lymphnode metastasis, TNM stage	/	/	Zhu et al. (2021)
6) circFOXM1	tumour size, TNM stage, nodular goiter	/	undifferentiated OS; DFS	Ye et al. (2020)
7) circBACH2	tumour size, lymphnode metastasis, TNM stage	diagnosing TC (AUC=0.882)	OS	Cai et al. (2019)
8) circ_0079558	tumour size, TNM stage			Zheng et al. (2021a)
9) circ_FNDC3B	tumour size, lymphnode metastasis, TNM stage	diagnosing TC (AUC=0.891)	OS	Wu et al. (2020a)
10) circ_0067934	tumour size, lymphnode metastasis, TNM stage	/	OS: an independent factor (RR=4.385)	Wang et al. (2019a)
11) circ_0001666	lymphnode metastasis	/	/	Qi et al. (2021b)
12) hsa_circ_102002	lymphnode metastasis, TNM stage	/	OS	Zhang et al. (2021c)
13) hsa_circ_0001018	lymphnode metastasis, TNM stage, distant metastasis	/	/	Luo et al. (2021)
14) hsa_circ_0008274	lymphnode metastasis, TNM stage, tumour infiltration	/	poor prognosis of TC	Ma and Kan (2021)
15) circPRMT5	lymphnode metastasis	/	/	Xue et al. (2020)
16) circ_0011058	lymphnode metastasis, TNM stage, nodular goiter	/	/	Zhang et al. (2021d)
17) circUBAP2	lymphnode metastasis, TNM stage	/	OS	Xiong et al. (2021b)
18) circPUM1	lymphnode metastasis, TNM stage	/	OS	Li et al. (2021d)
19) hsa_circ_0002111	lymphnode metastasis, TNM stage	diagnosing TC (AUC=0.833)	/	Du et al. (2021)
20) circZFR	lymphnode metastasis, TNM stage, extrathyroidal extension	/	OS	Wei et al. (2018)
21) hsa_circ_0058124	/	diagnosing TC (AUC=0.674)	/	Shi et al. (2020)
22) circ_RAPGEF5	/	diagnosing TC (AUC=0.7684)	/	Shi et al. (2020)
23) hsa_circ_0011290	/	/	OS	Hu et al. (2020b)
24) hsa_circ_0102272	TNM stage, histological grade, lymph node metastasis	/	hsa_circ_0102272 high expression was correlated with poor OS and PFS	Liu et al. (2020b)
25) hsa_circ_0124055	tumour size, TNM stage, histological grade, lymphnode metastasis	hsa_circ_0124055 distinguish TC (AUC=0.836), it combined with hsa_circ_0101622 provide diagnostic value (AUC=0.911)	OS	Sun et al. (2020b)
26) hsa_circ_0101622	tumour size, TNM stage, histological grade, lymphnode metastasis	diagnosing TC (AUC=0.805)	OS	Sun et al. (2020b)
27) circPVT1	tumour size, TNM stage, lymphnode metastasis	/	/	Zeng et al. (2021)
28) hsa_circRNA_007148	lymph node metastasis	diagnosing TC (AUC=0.846)	/	Ren et al. (2018)
29) circ_0059354	TNM stage, lymph node metastasis			Li et al. (2022)
30) circ_0067934	tumour size, tumour stage, lymphatic metastasis	/	/	Dong et al. (2022)
31) circ_0000144	tumour size, TNM stage, lymph node metastasis	/	/	Fan et al. (2020)
32) circRNA NRIP1	TNM stage	/	/	Li et al. (2021a)
33) hsa_circ_007293	lymphnode metastasis, TNM stage	/	/	Lin et al. (2021)
34) circ_0000644	tumour size, lymphnode metastasis	/	/	Nie et al. (2022)
35) circ-PRKCI	lymph node metastasis and recurrence	/	/	Liu et al. (2021b)
36) hsa_circ_0058124	advanced TNM stage, tumour size, extrathyroidal extension, lymph node metastasis, and distant metastasis	/	/	Yao et al. (2019)
37) circRNA UMAD1	side location, capsular invasion, vascular invasion, lymphnode metastasis, T stage, multifocality	diagnosing PTC with LNM (AUC=0.718)	/	Yu et al. (2020)
38) circRNA_103598	tumour size, TNM stage, metastasis status	diagnosing PTC (AUC=0.9465)	OS	Zhang et al. (2020b)
CircRNAs that are downregulated (↓) in thyroid cancer samples compared to control				
1) circHACE1	tumour size, lymphnode metastasis, TNM stage	/	/	Li et al. (2021b)
2) hsa_circ_0137287	tumour size, lymphnode metastasis, TNM stage	diagnosing TC (AUC=0.897); predicting extrathyroidal	/	Lan et al. (2018a)
3) circ_ITCH	lymphnode metastasis, TNM stage	/	/	Wang et al. (2018b)
4) hsa_circ_IPCEF1	lymphnode metastasis	diagnosing TC (AUC=0.801)	/	Guo et al. (2021)
5) combination of circRAPGEF5 and hsa_circ_0058124	no significant associations (such as age, gender, multifocality), correlate with lymphnode metastasis, TNM stage, distant metastasis	diagnosing TC (AUC=0.807)	/	Shi et al. (2020)
6) circ_0015278	extrathyroidal invasion, pTstage, pN stage, pTNM stage, a reduced relapse	diagnosing TC (AUC=0.903)	prolonged DFS: an independent factor	Ding et al. (2021a)
7) circNEURL4	lymphnode metastasis, TNM grade	/	OS	Ding et al. (2021b)
8) hsa_circRNA_047771	*BRAF* ^*V600E*^ mutation, lymph node metastasis, TNM stage	diagnosing TC (AUC=0.876)	/	Ren et al. (2018)

Abbreviations: OS: Overall survival; DFS: Disease-free survival; PFS: Progressive-free survival; RR: Relative risk; HR: Hazard ratio; pN: pathological node; pTNM: pathological tumour-node-metastasis.

CircRNAs have been reported to be significantly associated with many clinicopathological characteristics in TC, including tumour size, histological grade, lymph node metastasis (LNM), distant metastasis, multifocality, extrathyroidal extension, invasion and recurrence (Table 2). For example, Ye et al. observed that circFOXM1 is significantly upregulated in PTC tissues and in TPC-1 and BCPAP cells and that circFOXM1 levels are associated with tumor size (*p* = 0.001), TNM stage (*p* = 0.002), LNM (*p* = 0.002), and nodular goiter (*p* = 0.009) (Ye et al., 2020). In contrast, circ-ITCH is downregulated in PTC tissues and cell lines, and its expression levels are significantly associated with LNM (*p* = 0.020), clinical stage (*p* = 0.022) (Wang M. et al., 2018). Similarly, hsa_circ_IPCEF1 is significantly decreased in both PTC tissues and blood, and its levels were positively correlated with LNM (*p* < 0.001) (Guo et al., 2021). Most studies have reported that there is no relationship between circRNA levels and gender in TC. It should be noted that female have higher incidence and favorable DTC outcomes than male (Zhang D. et al., 2018).

Finally, defining a precise prognosis for TC patients is essential for physicians to formulate the best treatment decisions. To further analyse the prognostic value of circRNAs in TC, we collected information from studies reporting survival information and evaluated the associations between circRNA expression levels and overall survival (OS), disease-free survival (DFS), and progression-free survival (PFS) (Table 2) (Jin et al., 2018; Lan et al. 2018a; Ren et al. 2018; Wang et al. 2018b; Wei et al. 2018; Cai et al. 2019; Yao et al. 2019; Wang et al. 2019a; Fan et al. 2020; Han et al. 2020a; Hu et al. 2020b; Liu et al. 2020b; Shi et al. 2020; Sun et al. 2020b; Wang et al. 2020b; Wu et al. 2020a; Xue et al. 2020; Ye et al., 2020; Yu et al., 2020; Zhang et al. 2020b; Chu et al., 2021; Ding et al. 2021a; Ding et al. 2021b; Du et al. 2021; Guo et al. 2021; Li et al. 2021a; Li et al. 2021b; Li et al. 2021d; Lin et al. 2021; Liu et al. 2021b; Luo et al., 2021; Ma and Kan, 2021;Qi et al., 2021b; Xiong et al., 2021b; Zeng et al., 2021; Zhang et al. 2021c; Zhang et al. 2021d; Zheng et al. 2021a; Zhu et al., 2021; Dong et al., 2022; Li et al. 2022; Nie et al. 2022). For example, Wang et al. observed that circ_0067934 was highly expressed in TC tissues, and Cox proportional hazards regression model analysis indicated that circ_0067934 expression level was independently associated with OS (RR = 4.385, 95%CI = 1.087–17.544, *p* = 0.038) (Wang H. et al., 2019). Ding et al. revealed that higher circ_0015278 expression was independently correlated with improved DFS (*p* = 0.026, HR = 0.529) and found that higher pathological tumour-node-metastasis stage was an independent factor of shorter DFS (*p* = 0.017, HR = 1.766), and tumour size (>4 cm vs≤ 4 cm) as independent factors of unfavourable OS in patients with PTC (*p* = 0.012, HR = 4.835) (Ding H. et al., 2021). Similarly, a study by Liu et al. identified that higher expression of hsa_circ_0102272 resulted in worse OS and PFS in patients (Liu et al., 2020b).

### CircRNAs as Potential Targets for TC

Several oncogenic and antioncogenic circRNAs have been discovered to regulate the initiation and development of TC (Supplementary Material S1). Overexpression or knockdown of related circRNAs might be an effective intervention strategy for TC progression. RNA interference (Wang L. et al., 2018; Cooper et al., 2018), CPISPR/Cas9 editing (Piwecka et al., 2017), plasmid transfection (Tatomer et al., 2017), and lentivitral vector infection (Wang M. et al., 2018; Ding W. et al., 2021; Li et al., 2021b; Sa et al., 2021) are methods that can be used to regulate circRNA levels (Figure 4). Small interfering RNAs (siRNAs) or short hairpin RNAs (shRNAs) that were designed to target the backspliced junction region of oncogenic circRNAs may suppress tumour growth and metastasis in patient-derived xenograft (PDX) mouse models (Zhang Q. et al., 2021; Chu et al., 2021; Zhang W. et al., 2021; Chen et al., 2021d; Wang W. et al., 2021). The synthesis and circRNA sequences were cloned into specific plasmid vectors for the production of lentiviral particles, which stably transfected TC cell lines and expressed the corresponding and desired circRNAs (Figure 4) (Wang M. et al., 2018; Ding W. et al., 2021; Li et al., 2021b; Sa et al., 2021). For example, Li et al. found that the circHACE1 sequence was cloned into the pLO5-ciR vector (Geenseed, Guangzhou, China) for the production of lentiviruses to stably transfect DTC cell lines and then acted as a tumour repressor (Li et al., 2021b). Exogenous circRNAs might be from the transfection of purified *in vitro* generated circRNAs or delivered by specific vectors containing DNA cassettes, designed for circRNA expression (Li J. et al., 2020). So far, exogenous circRNAs have been successfully loaded into nanoparticles for targeted therapy due to the specific advantages of nanoparticles, such as reduced toxicity and precise targeting (Figure 4) (Aikins et al., 2020). Additionally, drugs or viruses can mediate anti-tumour effects through individual circRNA or circRNA-associated axes (Hou et al., 2018; Zhang S. et al., 2020). Some circRNAs mentioned above, such as circEIF6 (Liu F. et al., 2018), circ_NEK6 (Chen F. et al., 2021), circ_0011058 (Zhang Z. et al., 2021), are related to chemoradiation resistance in TC. Therefore, targeting circRNAs may be important for treating tumour resistance clinically and provide a new approach for TC treatment.

## Approaches for circRNA Studies and Future Perspectives

To better study the biological functions and applications of circRNAs, numerous circRNA-associated public databases (e.g., CircBase and Circ2Traits) have been developed to facilitate circRNA analyses (Ghosal et al., 2013; Glažar et al., 2014; Chen L. et al., 2021). Other databases and their common uses are listed in Supplementary Material S4. In addition, numerous approaches (e.g., GBDTCDA, iCDACMG and SGANRDA) have been proposed to find circRNA-cancer association (Lei and Fang, 2019; Wang L. et al., 2021; Xiao et al., 2021), which will contribute to elucidating the pathogenesis mechanisms and unveiling new insights for tumour diagnosis and targeted therapy. Furthermore, many bioinformatics tools (e.g., Find_circ, CIRI and CIRCexplorer pipelines) have been developed to recognise circRNAs by identifying the back-spliced junction (BSJ) reads (Memczak et al., 2013; Gao et al., 2015; Ma X.-K. et al., 2021). As a novel and increasingly popular research area, the bioinformatics toolboxes for circRNAs discovery and analysis remain in their infancy. The basic work of circRNA research need to be improved, such as establishing high quality dtabases, developing rapid and potent detection tools, and confirming the unified standard for detection methods.

CircRNAs were once considered the waste of error splice; however, recent studies have explored the comprehensive expression patterns of natural circRNAs and then screened and validated them in tumour (Liu et al., 2017). In addition, researchers have designed engineering circRNAs and their regulators for potent and durable protein expression *in vitro* (Wesselhoeft et al., 2018; Qi et al., 2021a). Artificial circRNAs function as miRNA and protein sponges have been the focus of research attention (Wang Z. et al., 2019; Schreiner et al., 2020). For example, Liu et al. constructed artificial circRNAs, which can suppress gastric carcinoma cell proliferation through sponging miR-21 (Liu X. et al., 2018). Jost et al. proved that artificial circRNAs inhibited viral protein production through sponging miR-122 (Jost et al., 2018). Although artifical circRNAs have many potential applications, they still face challenges due to the immunogenicity (Liu C.-X. et al., 2019). Liu et al. first revealed that synthesised circRNAs without extraneous fragments exhibited minimal immunogenicity and inhibiton related to PKR overreaction (Liu C.-X. et al., 2022). Qu et al. first reported a circRNA vaccine that encodes the trimeric receptor-binding domain of the SARS-CoV-2 spike protein (Qu et al., 2021) and elicits potent neutralizing antibodies and T cell responses, providing robust protection against SARS-CoV-2 (Qu et al., 2022). However, the immunogenicity of *in vitro* transcription-produced circRNAs is a potential concern and the safety of circRNA vaccines awaits further investigation (Liu L. et al., 2022).

Although great progress has been made in identifying circRNAs, the exact mechanisms of circRNA biogenesis and functions in TC remain largely unexplored. First, does circRNA actually circular? Sun et al. first suggested that circRNAs might not have a simple ring structure but contain a double-stranded structure, thus facilitating circRNAs export to the cytoplasm and making them more easily degraded (Sun et al., 2021). Second, how do circRNA decay? Some circRNAs are degraded by endonucleases (e.g., RNase P) in a primary sequence-dependent manner (Hansen et al., 2011; Park et al., 2019), another mechanisms (e.g., UPF1 and G3BP1) are associated with structure-mediated RNA decay (Liu C.-X. et al., 2019; Fischer et al., 2020). However, the detailed process is largely unknown. It will be essential to elucidate which endoribonuclease opens the closed loop of these circRNAs, how circRNAs are degraded by extracellular or intracellular signals, and what other factors contribute to structure-mediated RNA decay (Guo Y. et al., 2020). Third, extracellular vesicles (EVs) and exosomes have been used as drug and functional RNA delivery vectors in cancer treatment (Yang Z. et al., 2020). EVs-derived RNAs are essential functional cargoes in reciprocal crosstalk within tumor cells and between tumor and stromal cells (Hu W. et al., 2020). In addition, EVs-derived circRNAs can enhance functional recovery in post stroke and may extend the therapeutic window for stroke (Yang L. et al., 2020). However, obstacles that need to be overcome towards clinical utilisation include upscaling of the EVs production and isolation process, and guidelines for appropriate storage (Elsharkasy et al., 2020). Forth, the mechanisms guiding circRNAs exosome assembly, lysosomal exocytosis and endocytosis are poorly understood. Although study clarify that exosomes contain transmembrane and membrane anchoring proteins, which enhance endocytosis (Kamerkar et al., 2017), more efforts are still needed to make the diagnostic and therapeutic potential of exosomes a clinical reality. Finally, knockdown of circFSCN1 and circ_Malat 1 can effectively prevent alloimmune rejection in heart transplantation (Zhang Y. et al., 2018; Wang B. et al., 2021). exosome-based delivery products can induce an early T cell response and initiate antitumor immune responses (Gilligan and Dwyer, 2017; Seo et al., 2018). However, there is no evidence that exosomal circRNAs contribute to preventing immunological rejection in tumour.

Although dysregulated circRNAs and their function contribute to TC initiation and progression, the underlying mechanisms remain poorly defined. First, researchers have proposed that a balance exists between circRNA generation, intracellular localisation, and degradation. Once this balance is tipped, circRNA becomes dysregulated (Li J. et al., 2020). Second, ceRNA hypothesis have been recognised as the most common mechanism for circRNAs to utilise their function, but the function of miRNA sponge still faces challenges (Thomson and Dinger, 2016). Few circRNAs harbour as many miRNA binding sites for a single miRNA as ciRS-7 (Hansen et al., 2013) and circZNF91 (Kristensen et al., 2018), and the abundance of many circRNAs is far lower than that of miRNAs, preventing them from achieving the miRNA sponge effect. In addition to the stoichiometric relevance between the miRNA-binding sites and the mRNA target sites of the miRNA need to be considered, Ago-CLIP/AgoIP and quantitative analysis of specific primers are also required to confirm the function of miRNA sponges. Third, recent study has clarified that the ciRS-7 is upregulated in stromal cells within the tumour microenvironment, but is absent in tumour cells, particularly in classical oncogene-driven adenocarcinomas (Kristensen et al., 2020). The spatial expression patterns of circRNAs at the single-cell level are crucial for understanding the function of circRNAs and advancing the discovery and development of biomarkers in the future. More than fifty clinical trails hve been registered on the website of *Chinese Clinical Trial Registry* and *National Library of Medicine*, thus highlight the important roles of circRNAs in human diseases (e.g., pancreatic cancer and COVID-19), but these functions are only the beginning.

Numerous studies investigated various DECs between thyroid tumours and the adjacent non-tumour tissues. Some circRNAs (e.g., hsa_circRNA_047,771) were associated with the *BRAF* ^*V600E*^ mutation (*p* < 0.05) in PTC (Ren et al., 2018). The presence of *BRAF* ^*V600E*^ mutation at PTC diagnosis is associated with aggressive tumour characteristics (*p* < 0.001) (Xing et al., 2013). Furthermore, *BRAF* ^*V600E*^ mutation may lead to a decrease in the therapeutic effect of radioactive iodine, resulting in treatment failure or recurrence (Ge et al., 2020). Targeting circRNAs related to *BRAF* ^*V600E*^ mutation may contribute to reducing the recurrence and improve the outcome of TC. A 5-years cohort study suggested that patients with thyroid nodules increased by ≥ 3 mm in only 8% of patients, and only 3.8% of patients developed nodal metastases (Ito et al., 2014). However, the overtreatment of TC has been recognized as an urgent issue. Many asymptomatic TC patients treated with surgery may suffer from permanent hypoparathyroidism and recurrent laryngeal nerve injuries, and need long-term hormone replacement therapy (Luster et al., 2014; Jegerlehner et al., 2017). Therefore, accurately identifying the circRNAs associated with TC helps in the diagnosis and treatment of TC.

## Conclusion

In summary, circRNAs constitute an emerging class of ncRNAs that play crucial roles in the regulation of gene expression by controlling miRNA and protein functions. With the broad applications of high-throughput sequencing technology and bioinformatics analysis in scientific research, the number of circRNAs with known functions is increasing. Notably, circRNAs mediate central biological functions including various physiological and pathophysiological processes, rendering them ideal candidates in the field of cancer research.

Our review discussed and summarised the emerging data and research progress on TC-associated circRNAs, and further highlighted individual circRNAs that may play oncogenic, anticancer, or sensitivity to chemoradiation regulating role in the tumourigenesis, metastasis and therapy resistance of TC by various molecular mechanisms. These circRNAs provide a new area of interest for developing TC diagnostics, prognostics, and therapies. Since the current understanding of circRNAs is basic, much research is required to reveal its regulatory mechanisms and subsequent biological functions in TC.

## Supplementary Material

Supplementary Figure S1 Network of circRNA-miRNA-mRNA interactions in thyroid cancer.

Supplementary Table S1 Biological functions and molecular mechanisms of circRNAs in thyroid cancer.

Supplementary Table S2 Network of circRNA-miRNA-mRNA interactions in thyroid cancer.

Supplementary Table S3 Databese for circRNA research.

## Glossary

TC: Thyroid cancer
circRNAs: Circular RNAs
RBPs: RNA binding proteins
PTC: Papillary thyroid cancer
DTCs: Differentiated thyroid cancers
EcircRNAs: Exonic circular RNAs
ciRNAs: Intronic circular RNAs
EIciRNAs: Exon-intron circular RNAs
MecciRNAs: Mitochondria-encoded circular RNAs
TricRNAs: Pre-tRNA circular RNAs
tRNA: transfer RNA
RNA Pol II: RNA polymerase II
ICSs: Intronic complementary sequences
QKI: Quaking
m6A: N6-methyladenosine
ceRNA: Competitive endogenous RNA
miRNA: microRNA
AGO2: Argonaute 2
YTHDF2: YTH domain-containing family protein 2
UPF1: Upstream frameshift 1
RNase H1: Ribonuclease H1
ncRNAs: Noncoding RNAs
LncRNA: Long noncoding RNA
DECs: Differentially expressed circRNAs
FC: Fold change
LNM: Lymphnode metastasis
EMT: Epithelial-mesenchymal transformation
AhR: Aryl hydrocarbon receptor
PDK: Pyruvate dehydrogenase kinase
AUC: Area under the receiver operative characteristic curve
ROC: Receiver operative characteristic curve
OS: Overall survival
DFS: Disease free survival
CI: Confidence interval
RR: Relative risk
HR: Hazard ratio
PDX: Patient-derived xenograf
BSJ: Back spliced junction
PKR: Protein kinase R.
