# Supplementary figures and images for: Function and Clinical Significance of Circular RNAs in Thyroid Cancer
Source: Front Mol Biosci. 2022 Jul 22;9:925389. doi: 10.3389/fmolb.2022.925389 (PMC9353217; doi:10.3389/fmolb.2022.925389)

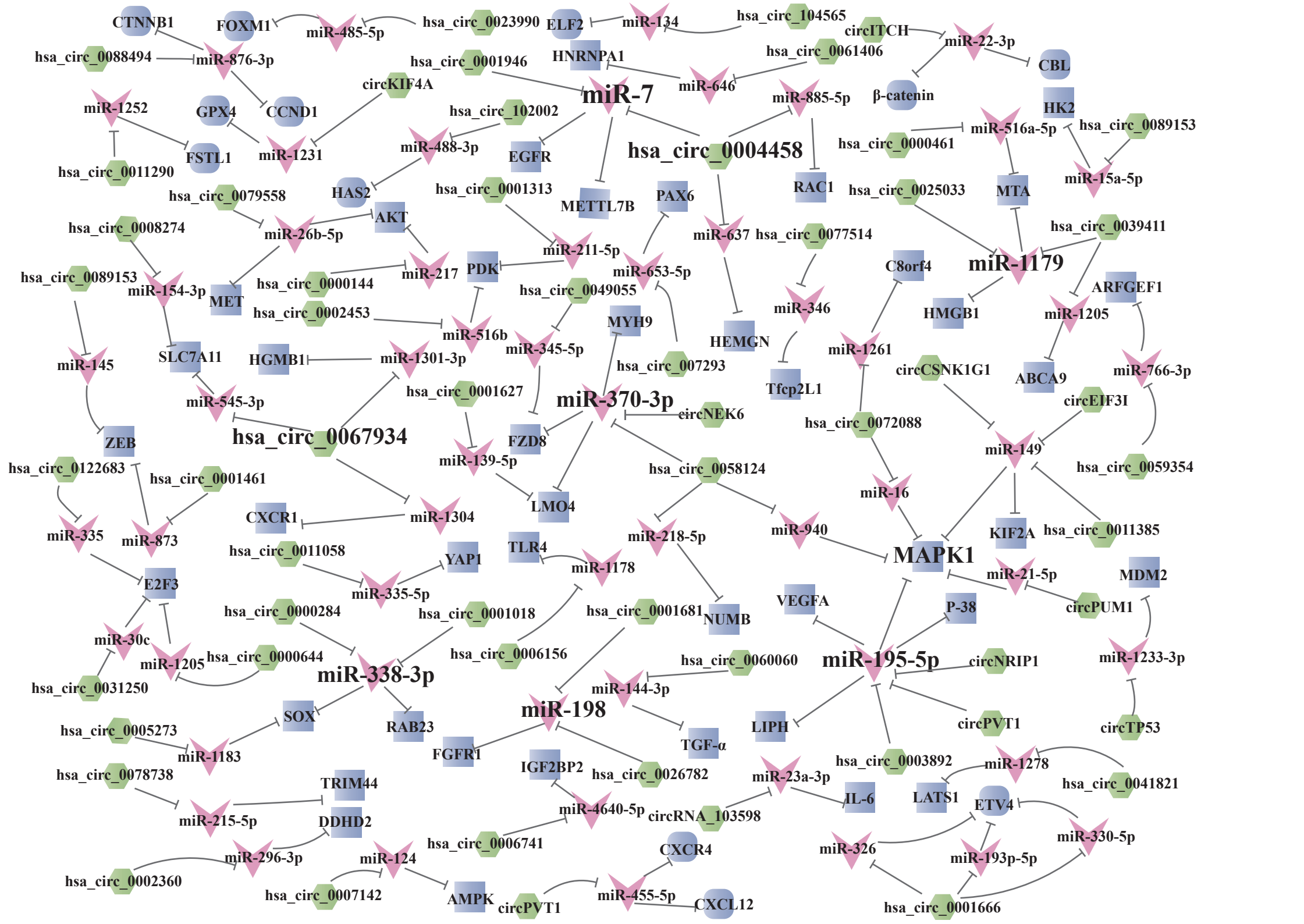

Supplement: Supplementary file 4 [file Image1.pdf]
